# Expression of the NSE,SP,NFH and DβH in normal and cryptorchid testes of Bactrian camel

**DOI:** 10.1590/1984-3143-AR2021-0087

**Published:** 2022-02-04

**Authors:** Ligang Yuan, Hua Wang, Hongzao Yang, Shaoyu Chen, Dapeng Yang, Yong Zhang

**Affiliations:** 1 College of Veterinary Medicine, Gansu Agricultural University, Gansu Key Laboratory of Animal Generational Physiology and Reproductive Regulation, Lanzhou, China.

**Keywords:** Bactrian camel, cryptorchidism, neuroendocrine markers, immunohistochemistry

## Abstract

Neuroendocrine substances play essential roles in regulating the normal physiological functions of testicles. The purpose of this study is to explore the localization and effects of four neuroendocrine markers (NSE, SP, NFH and DβH) in normal and cryptorchid testes of Bactrian camels using western blotting, transmission electron microscopy, immunohistochemistry, and immunofluorescence methods. The results showed that cryptorchidism caused a reduction in layers of spermatogenic epithelium and decreased glycogen positivity in the basement membrane. The ultrastructure revealed that macrophages were always found around the Leydig cells, crowded with swelling mitochondria in cryptorchidism. Expression of NSE in the Leydig cells of cryptorchidism was significantly weakened compared to that in the normal group(*p*<0.01). We found that SP was always distributed along the nerve fibers in normal testes and was expressed in the Leydig cells of cryptorchidism. However, expression of NFH in the cryptorchidic tissue was strongly positive in the spermatogenic epithelium, with limited expression in Leydig cells and no expression in peritubular myoid cells. Therefore, the expression of DβH in the Sertoli cells was comparatively strong in both the normal and cryptorchidism groups. NFH and DβH expression was significantly increased in the cryptorchidism group compared with the normal group (*p*<0.01). These findings indicated that the underdeveloped seminiferous epithelium and pathological changes in cryptorchid tissue in Bactrian camels were potentially related to a disorder in glycoprotein metabolism. Our results suggest that NSE and SP could help judge the pathological changes of cryptorchidism. The present study provides the first evidence at the protein level for the existence of NFH and DβH in Sertoli and Leydig cells in Bactrian camel cryptorchidism and provides a more in-depth understanding of neuroendocrine regulation is crucial for animal cryptorchidism.

## Introduction

Gametogenesis in mammals is a very complex process in which paracrine and autocrine mechanisms in addition to the hypothalamic-pituitary-testicular endocrine axis influence are intimately involved in complicated feedback loops (Plant, 2015; Khanehzad et al., 2021). Leydig cells originate in the adult testis by differentiation from embryonic neuroectoderm precursor cells and mesenchymal cells and express a variety of neural and neuroendocrine markers (Davidoff et al., 2002; Davidoff et al., 2009).Studies have suggested that in addition to steroid hormones the Leydig cells of different rodents and humans produce and possess several neuronal markers such as 5-hydroxytryptamine(5-HT), neural cell adhesion molecule(N-CAM), and microtubule-associated protein (MAP-2) catecholamine and norepinephrine protease (Krobert et al., 2001; Müller et al., 2006; Davidoff et al., 2009).The distribution and localization of PGP9.5,neuropeptide Y, neuron-specific enolase (NSE), substance P(SP), dopamine-β-hydroxylase (DβH), and tyrosine hydroxylase-related peptides in the testicles of mammals, such as cats (Suburo et al., 2002), donkeys (Wrobel and Moustafa, 2000), ruminants (Maddocks et al., 1995), dromedaries (Saleh et al., 2002) and humans (Gong et al., 2009) were studied by immunohistochemistry. Results showed that such neuroendocrine substances play essential roles in regulating the normal physiological functions of the testicles. Previous researches have shown that the expression of NSE, SP and NFH (High Neuro-filaments ) in the testicular tissues of adult rats is regulated by serum sex hormone concentrations (Ortega et al., 2004). The occurrence of numerous marker substances, such as SP, NFP-200, NSE and DβH characterized human Leydig cells as new members of the "diffuse neuroendocrine system"or the family of paraneurons (Davidoff et al., 2002; Plant, 2015). Normal levels of neuroendocrine markers are indispensable for spermatogenesis, and malfunction of one or more of the neuroendocrine regulated processes within the testis is closely related to the occurrence of sterility (Huo et al., 2010; Yuan et al., 2015; Manfredi-Lozano et al., 2018). However, the neuroendocrine nature of camel infertility is not well characterized. Here, we present histochemical results and the distribution characteristics of four neuroendocrine markers (NSE, SP, NFH and DβH) in the cryptorchidism of camels. These results are necessary to clarify the capacity of Leydig cells to synthesize these substances and establish their functional significance in reproduction.

The Bactrian camel (Camelus Bactrianus) is an essential domestic animal in some of the desert and semi-desert areas of the world. Its ability to reproduce in very poor living conditions is related to its unique breeding mechanism. Cryptorchidism is reproductive system disease that causes sterility in camels. A few studies have reported the pathological characteristics of testes in infertile male Bactrian camels, such as abnormalities in the seminiferous tubule and the decrease in the synthetic ability of Leydig cells was a meaningful index for the further research of the steroid biosynthesis (Yuan et al., 2016; Yuan et al., 2017b). However, there is no detailed report about the neuroendocrine characteristics of cryptorchid testes in Bactrian camels. Additionally, we have compared the expression of the PGP9.5 and neuropeptide Y in Leydig cells between the normal and cryptorchid testes of Bactrian camels, and reported for the first time the role of neural markers on Bactrian camel infertility (Yuan et al., 2015). Based on these findings, the aim of this study was to compare the distribution of NSE, SP, NFH and DβH in Leydig cells from the normal and cryptorchid testicular tissues of Bactrian camels. We also investigated the microstructural characteristics and markers of Leydig cells to explore the changes of the Leydig neuroendocrine characteristics, suggest that the young adult cryptorchid camels are innervated by NFH and DβH, which may serve as significant endocrine markers for testicular functions at this age. The expression of SP was significantly decreased in cryptorchidism provide a morphological basis for further research on the roles in testes descent. The innervation of Bactrian camels testis by cholinergic and adrenergic nerves present are summarized in this study and include evidence of indirect, as well as the rare direct neuroendocrine character of cryptorchidism Bactrian camels.

## Materials and methods

All protocols involving the use of animals were performed under the approved Guidelines for Animal Experiments of Gansu Agricultural University and were approved by the Animal Experimental Ethical Inspection Committee of Gansu Agricultural University (NO. GASU-Eth-VMC-2018-010)

### Experimental materials

#### Experimental animals

A total of 11 male unmated about two-year-old Bactrian camels were collected in winter from breeding farmers in Laohe County, Wuzhong City, Ningxia Hui Autonomous Region. These home-bred animals were divided into two groups: the normal (n=5) and cryptorchidism groups (n=6) for comparative analyses. The cryptorchid testes were located in the abdominal cavity (Intra-abdominal cryptorchidism, the bilateral testicles fail to descend into the scrotum). Tissue specimens were collected by orchiectomy.

The samples were treated according to different test schemes. Fresh testes were processed into small pieces and then divided into three samples. One sample was frozen in liquid nitrogen for western blotting hybridization reaction, while the other sample was fixed with 4% paraformaldehyde solution for histochemical analysis, and the third fixed in glutaraldehyde for transmission electron microscopy observation.

#### Main reagents

All antibodies were purchased from commercial suppliers. Rabbit polyclonal antibody NSE (bs-10445R-HRP,anti- NSE/HRP), SP(bs-0065R-HRP, anti-SP/HRP), NFH (bs-10680R-HRP, anti-NFH/HRP) and DβH (bs-0596R-HRP, anti-DβH /HRP), anti-rabbit IgG antibody(bs-0295G-AF488) and DAB chromogenic kit(ZLI-9018) were purchased from Beijing ZSGB-BIO Co., Ltd., Beijing, China, Immunohistochemical staining kit(SP-0023), produced by ZYMED Laboratories Co., Ltd., San Diego, USA, purchased from Jiamay Biotech Co., Ltd., Beijing, China. Furthermore, other chemicals were obtained commercially and of reagent grade.

### Experimental methods

#### H&E staining, histochemical staining, and observation of tissue samples

Testicular tissue samples (1×1× 0.6 cm) were fixed with paraformaldehyde and rinsed in running water for 24h before conventional gradient ethanol dehydration. Afterward, they were made transparent with xylene, embedded in paraffin, and 5μm serial sections were cut every fifths section was mounted and stained either with standard hematoxylin and eosin(H&E)to examine the general morphology, followed by Alician blue(AB pH=2.5,30 min)and periodic acid Schiff reaction(PAS)and examined for the presence of positive staining cells, the acidic mucin and proteoglycan were blue, with the nuclei being reddish (Culling, 1974).

#### Transmission electron microscopic technique

The testes were cut into 0.1cm×0.1cm×0.1cm,fixed in the2.5% glutaraldehyde in 0.1M phosphate-buffered saline(pH=7.4)or 48h,and then postfixed in 1% osmium tetroxide at 4°C for 1h.The pieces were dehydrated with a graded ethanol series, and then embedded in epoxy resin (Epon812,American).Ultrathin sections were cut, stained with uranyl acetate and lead citrate, and then examined under a JEM-100CX electron microscope (NEC, Japan).

#### Immunohistochemical staining

The streptavidin-peroxidase method was used for immunohistochemical staining. The paraffin sections were routinely dewaxed and differentiated by gradient ethanol;3% H_2_O_2_ in methanol solution was used to block peroxidase for 10min.Normal goat serum albumin was incubated for 15min; 50μL of rabbit polyclonal anti-mouse NSE (bs-10445R), SP (bs-0065R), NFH (bs-10680R) or DβH (bs-0596R) antibodies were diluted at 1:400 and added dropwise to each section separately. After incubation at 37°C for 2h and washing by PBS shaking, 50μL of biotinylated goat anti-rabbit IgG working solution was added dropwise to each section, followed by the addition of 50μL of horseradish-labeled streptavidin working solution. The sections were then incubated at 37°C for 15min.Freshly prepared DAB substrate soluteion was then added for microscopic examination, followed by routine dehydration, making transparent, and mounting. Positive cells displayed as brown-yellow, with nuclei showing as blue after counterstaining. The negative control group was stained with 0.01mol·L-1 of PBS instead of the primary antibody.

#### Immunofluorescence staining

Green immunofluorescence staining :Paraffin sections of testicular tissues were prepared following the same procedure as demonstrated in section 1.2.1.Slice thickness was 5μm,high-pressure antigen retrieval was performed,30g/L H_2_O_2_ aqueous solution was added to block peroxidase for 10min,Sections were incubated with goat serum albumin for 15min.A volume of 50μL of primary antibody (rabbit polyclonal anti-NSE, anti-SP, anti-NFH and anti- DβH)was added dropwise at a dilution ratio of 1:1,000.For the negative control group,0.01mol.L-1 phosphate buffer solution buffered saline(PBS)was used as the substitute for the primary antibody. Incubation was carried out for 2h at 37°C before the fluorescent secondary antibody was added dropwise. Alexa Fluor488-labeled goat anti-rabbit IgG antibody (1:1,000,bs-0295G-AF488) was used as the secondary antibody. After incubation at 37°C for 2h, observations were made directly under the microscope. Positive cells displayed fluorescent green and the immunofluorescence images were collected by ECHO fluorescence microscope (REVOLVE RVL-100-G).

#### Western blot analysis

Total protein was extracted from testicular tissue was extracted.100mg of liquid nitrogen-preserved testicular tissue was weighed and crushed. Radioimmunoprecipitation assay buffer containing phenylmethylsulfonyl fluoride was added proportionally. The mixture was then homogenized with a glass homogenizer until fully lysed, and then subjected to hypothermal centrifugation at 14,000×*g* for 5min.The supernatant was transferred to another tube, and reagent with half of the volume of the solution was used to dissolve the precipitate. After standing still at 25°C for 30 minutes, the mixture was centrifuged at 4°C and 19,575×*g* for 20min.The supernatant was analyzed for protein concentration according to the instructions of the BCA protein assay kit (Beyotime Biotechnology Research Institute),dispensed, and stored at -70°C for use.

The protein was extracted and loaded following the routine procedure: the protein was separated by polyacrylamide gel electrophoresis (SDS-PAGE); 5% stacking gel and 12% separating gel were prepared. 30μg of protein was loaded to the gel sample well for electrophoresis, and the protein in the gel with the target band was subjected to wet transfer to the membrane. The membrane was incubated with primary antibody NSE, SP, NFH, or DβH (1:1,000) at 4°C overnight, and then was washed by Tris-buffered saline+Tween 20(TBST). The horseradish peroxidase-labeled cow anti-rabbit IgG was used as the secondary antibody for incubation for 2h at 25°C, and TBST was used to wash the membrane three times for 10min each. The polyvinylidene fluoride membrane was then subjected to color development. The chemiluminescent substrate solutions A and B were mixed at a ratio of 1:1 and were allowed to react at 25°C. The transfer membrane was photographed for analysis.β-actin was used as the internal reference.

### Measurement and statistical analysis

Tissue sections were imaged using a Nikon Eclipse 80i microscope camera system. Leydig cell characteristic index: ten sections were randomly selected from each group, and six non-repeating fields were randomly selected from each section (bar=20μm, 400×). The transverse and longitudinal diameters of Leydig nuclei, as well as the mean area of Leydig nuclei in the triangular and quadrangular mesenchymal tissues of each field were randomly counted (n=40). Statistical analyses were performed by Image Pro Plus 6.0 software.

The intensity of immunofluorescence results was scored as follows: -: no positive expression; +/-: occasional positive expression; +: positive expression; ++: medium-intensity positive expression; +++: strong positive expression; ++++: high-density strong positive expression (Amadio et al., 2007)

The sections were imaged using a NIKON ECLIPSE 80i microscope camera system. Five sections were selected randomly from each group for immunohistochemical staining. Six non-repetitive fields (bar=20μm, 400×) were randomly selected for each section. The mean positive signal intensity and positive area of each field were statistically analyzed by Image Pro Plus 6.0 software to evaluate the average light absorbance. A total of 30 statistical data were collected for each group, and the results were expressed as mean±standard deviation (mean ± SD).SPSS15.0 software was used for statistical analysis, and the expression difference of NFH, SP, NSE and DβH between normal and cryptorchid testes was analyzed by one-way analysis of variance. Paired t-tests were carried out, and the level of statistical significance was set at *p*<0.05.

The western Blot expression band was first selected, the gray curve of which was then analyzed using Image J 1.48.The area under the peak was calculated as the band density value. The density of β-actin was taken as the base value, and the relative densities were obtained by comparing the densities of NSE, SP, NFH, or DβH expression bands in the normal testis group (Simply marked as N-NSE, N-SP, N-NFH, or N-DβH) and the cryptorchidism group(Simply marked as C-NSE, C-SP, C-NFH, or C-DβH).The relative density values were then statistically processed by SPSS 21.0 statistical software. All data were expressed as mean±standard deviation. The differences between variables was analyzed by t-tests difference between variables were analyzed using paired t-tests, and the level of statistical significance was set at p< 0.05.

## Results

### Comparison of the histochemical characteristics between normal and cryptorchid testes of Bactrian camels

**Figure 1:**
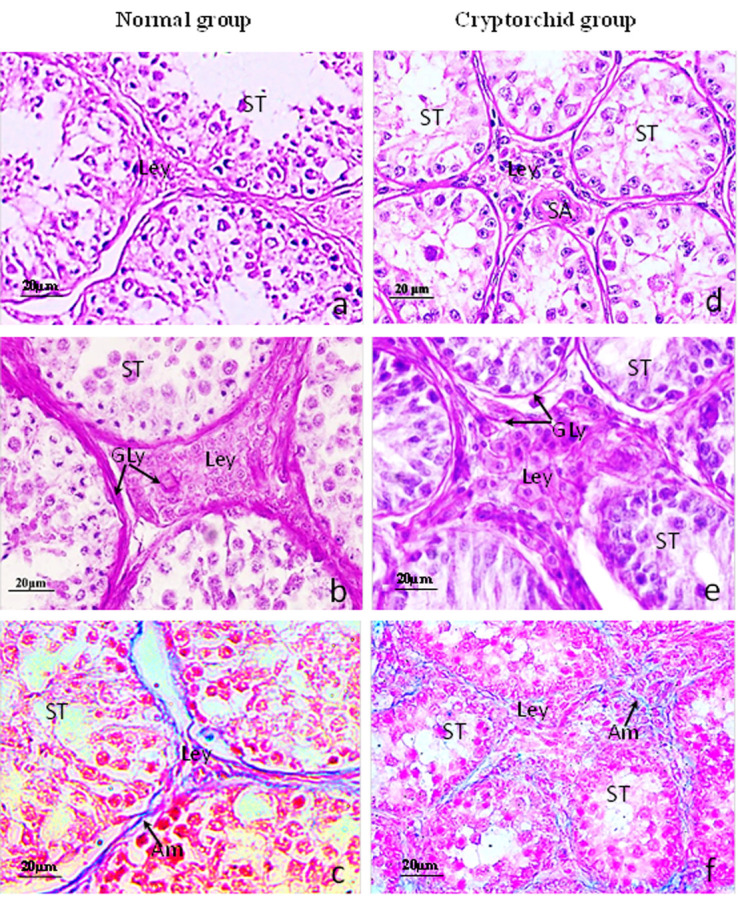
Comparison of the histochemical characters in normal and cryptorchid testes of Bactrian camels. a-c: Normal Bactrian camels; d-f: Cryptorchid Bactrian camels; (a,d): stained with H&E; (b,e): stained with PAS; (c,d): stained with AB. (a) Revealed that the seminiferous epithelium of Bactrian camels was developing well in the normal group; (b) The glycogen was detected by PAS staining and distinct purple-red glycogen-positive band in the lamina propria and interstitial capillary walls; (c) The positive reaction of AB staining were present clearly throughout the basement membranes and the wall of interstitial blood vessels in the normal testes; (d) The cryptorchidism causes a reduction in layers of spermatogenic epithelium; (e) Decreased PAS positive in the basement membrane of the seminiferous tubule; (f) The positive reaction of AB was almost invisible in the interstitial tissue of the cryptorchid group.

As shown in Figure 1, H&E staining revealed that the seminiferous epithelium in normal testes of Bactrian camels was developed well with seminiferous cells located on both sides of Sertoli cells and round, oval, or irregular-shaped Leydig cells of considerably large size (Fig. a). We performed a histochemical analysis to identify the cellular changes underpinning the changes in cryptorchidism. We detected glycogen by PAS staining and distinct purple-red glycogen-positive bands in the lamina propria and interstitial capillary walls were observed (Fig. b); Acid mucins was also clearly visible in the interstitial capillary wall and lamina propria as demonstrated by AB staining (Fig. c); Examination of the H&E images revealed that cryptorchidism causes a reduction in layers of spermatogenic epithelium, the Leydig cells lacked distinct structural characteristics, and the nucleus was round, elliptical, or irregular, with relatively large cell bodies (Fig. d); We next examined whether r a decrease in glycoprotein metabolism caused the reduction in seminiferous tubule cross-section. PAS staining of the cryptorchid testes showed positive expression in the basement membranes and the wall of interstitial blood vessels (Fig. e), while the AB blue positive band was almost invisible in the interstitial tissue of the cryptorchid group (Fig. f).

Acidic mucoprotein(Am); Blood capillary(BC); Glycogen(Gly); Leydig cell(Ley); Small artery(SA); Sertoli cells (Sc); Seminiferious tubule(ST); Scale bar=20μm

Statistical analyses indicated that the transverse and longitudinal diameters of the nuclei in Leydig cells of cryptorchid Bactrian camels were significantly increased compared to those in normal testicles (*p*< 0.05). The average areas of the Leydig cells were significantly larger in cryptorchid testes than in normal testes (*p*<0.01, Table 1).

**Table 1:** The characteristics index of the leydig cells in Bactrian camel normal testis and cryptorchidism (mean±SD n=40).

Significant differences with normal group *p<0.05; ** *p*<0.01.

**Table:** 

**Group**	**The transverse diameter of the leydig cell nucleus(μm)**	**The Longitudinal diameter of the leydig cell nucleus(μm)**	**Average area of leydig cells nucleus(μm^2^)**
Normal group	12.217±2.745	12.864±2.282	124.428±18.539
Cryptorchid group	9.612±2.263*	10.156±2.689*	96.703±14.533**

### Comparison of the ultrastructure of Leydig cells between normal and cryptorchid Bactrian camels

**Figure 2:**
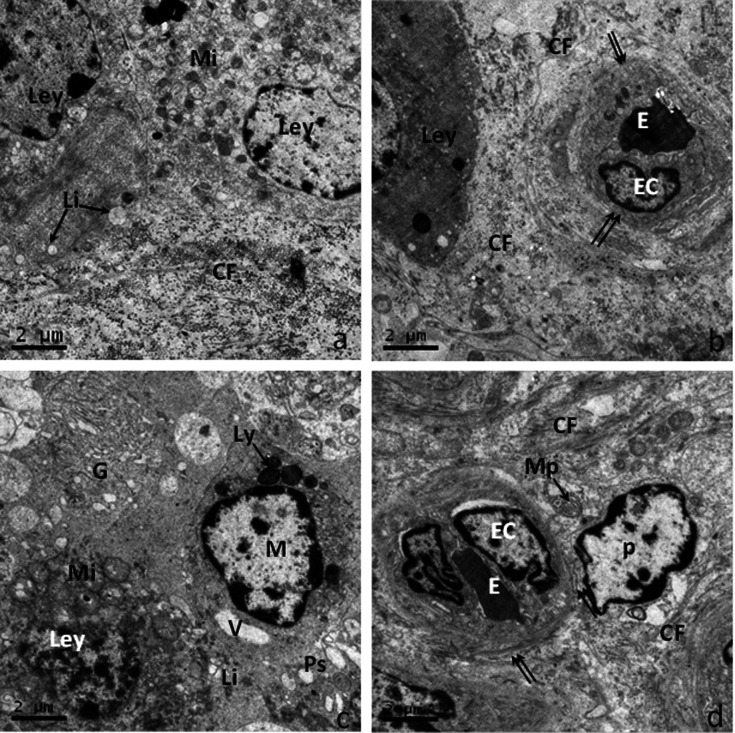
The ultrastructure of Leydig cell in the normal and cryptorchid testes of Bactrian camels. a-b: Normal Bactrian camels; c-d: Cryptorchid Bactrian camels; (a) Elaborately developed organelles of the Leydig cells in the normal testis; (b) The Leydig cells and blood capillaries in the interstitial tissue of the normal group; (c) The macrophages were always found around the Leydig cells in cryptorchidism; (d) The epithelial basal lamina of the capillary vessel was clear and scattered with microfino-cytotic vesicles in cytoplasm. Collagen fiber (CF); Erythrocyte (E); Endothelial cells (Ec); Golgi apparatus (G); Leydig cell (Ley); lysosomes (Ly); Macrophages (M); Mitochondrion (Mi); Lipid droplet (Li); Microparticles (MP); Pericyte (P); Pseudopod (Ps); Vacuole(V).scale bar=2μm.

Electron micrographs showed Leydig cells of the normal testis with the typical features of granular reticulum in the cisternae continuity in directly with this type of agranular reticulum. The granular reticulum was also sparse, the Golgi apparatus and mitochondria were dispersed, and lipid droplets were not evident (Figure 2a). Furthermore, collagen fibers were distributed around the interstitial capillaries, which revealed endothelial cells with flat nuclei and transparent basement membranes in normal testicular tissue (Figure 2b). The villi protrusions of the Leydig cell were unobvious and the swelling mitochondria and indistinct reticulum crowded in the cytoplasm which also consisted of few lipid droplets.Moreover,the macrophages around Leydig cells exhibited obvious pseudopodia, but many lysosomes of different sizes and residual bodies were observed in the cytoplasm (Figure 2c). The capillary vessel was smaller with mostly clear epithelial basal lamina, and scattered particles were observed between adjacent microvessel endothelial cells, while perivascular collagen fibrils were more affluent in the cryptorchid group (Figure 2d).

### Comparisons of the distributions of NSE, SP, NFH and DβH in normal and cryptorchid testes of Bactrian camels

**Figure 3:**
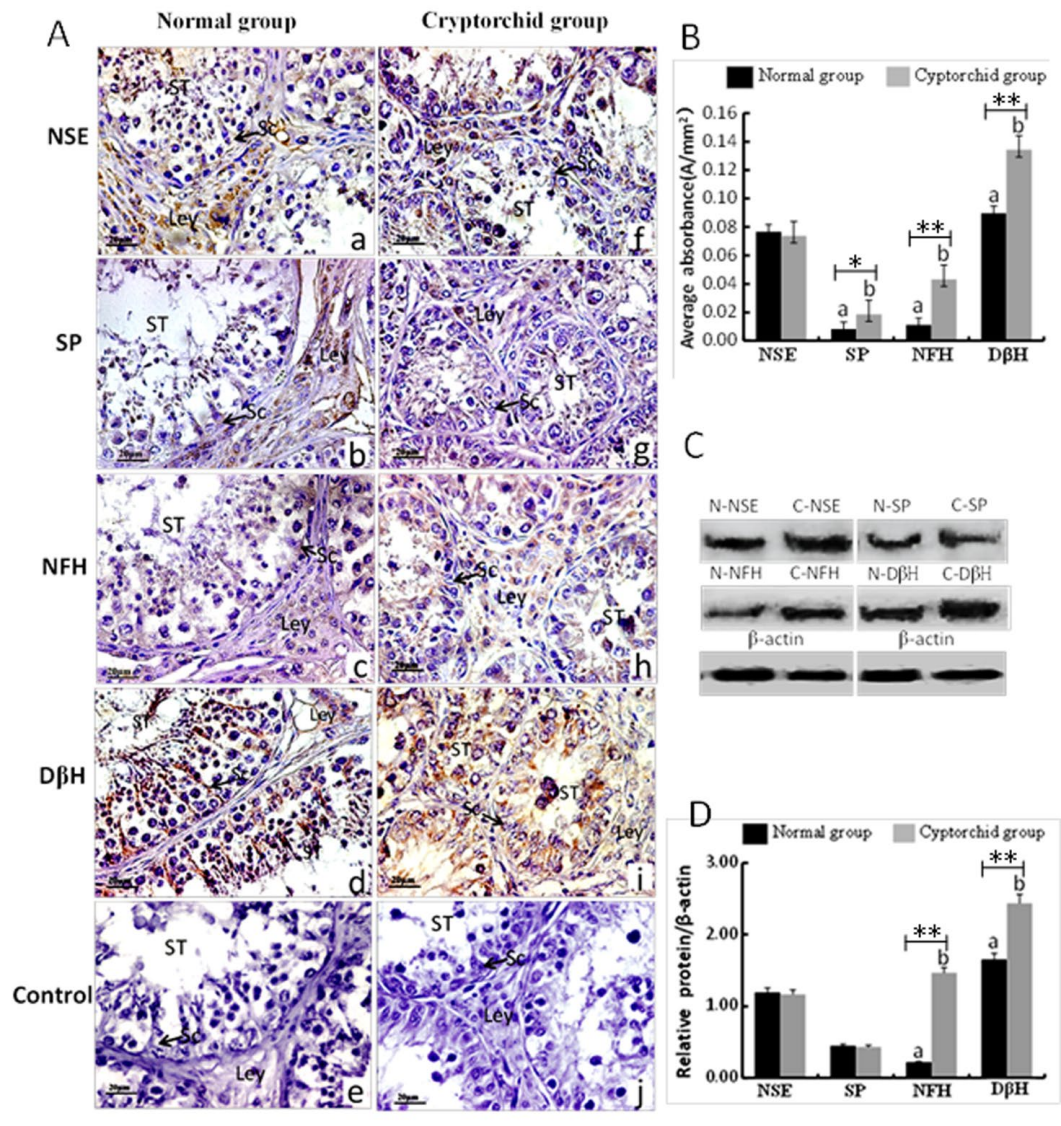
Distribution (A,B) and protein (C,D) levels of NSE,SP,NFH and DβH levels in normal and cryptorchid testes of Bactrian camels. (A) Tissue sections were stained with specific antibody and counterstained with hematoxylin. Positive signals appeared brown in color,and counterstaining background appeared blue in color. a-e: normal testicular tissues; f-j: cryptorchid testicular tissues;a and f:stained with NSE polyclonal antibody;b and g:stained with SP polyclonal antibody; c and h: stained with NFH polyclonal antibody; d and i: stained with DβH polyclonal antibody; e and j: negative control (no significant immunoreactivity was observed when normal rabbit serum instead of the primary antibody); Leydig cells (Ley); Sertoli cells (Sc); Seminiferious tubule(ST).scale bar=20μm. (B) Average absorbance of neuroendocrine markers (NSE,SP,NFH and DβH) in normal and cryptorchid testicular tissues of Bactrian camels. Data are expressed as fold change. The data are presented as mean ± SD.value from 40 statistical figures. Different letters indicate significant differences of the expressions in the testes of the Bactrian camel cryptorchidism compared with the normal groups (*p<0.05,** *p*<0.01). (C and D) Relative protein levels of NSE,SP,NFH and DβH in normal and cryptorchid testes of Bactrian camels. The density of β-actin was taken as the base value, and the relative densities were obtained by comparing the densities of NSE,SP,NFH or DβH expression bands in the normal testis group (Simply marked as N-NSE,N-SP,N-NFH or N-DβH)and the cryptorchidism group (Simply marked as C-NSE, C-SP, C-NFH or C-DβH). Data are expressed as fold change.The data are presented as mean±S.E.M. value from 12 statistical figures. Different letters indicate significant differences in the expressions of same neuroendocrine marker between the groups (**p<0.01).

In normal tissues (Figure 3A a-d), the expression of NSE was primarily observed in Leydig cells and vascular endothelial cells,not in seminiferous epithelial cells (Figure 3A a), while the prominent SP immunopositive nerve fibers in the interstitial tissue surrounding the Leydig cells (Figure 3A b); In addition,almost no NFH expression was observed in testicular tissues (Figure 3A c),whereas DβH was positively expressed in Sertoli cells, but not in seminiferous cells,Leydig cells or the surrounding tissues (Figure 3A d).No negative control had a positive reaction (Figure 3A e).Compared to normal testes, the expressions of NSE was blatant in Sertoli cells, peritubular myoid cells, and Leydig cells in cryptorchid tissues (Figure 3A f), but SP expression in the seminiferous epithelium and Leydig cells was significantly decreased (Figure 3A g), and NFH was clearly expressed in Leydig and seminiferous cells (Figure 3A h).In cryptorchid tissues, DβH was highly expressed in cytoplasm-rich primary spermatocytes, with occasional expression observed in atypical nuclear-shaped Sertoli cells (Figure 3A i).No expression of endocrine markers was detected in the negative control group (Figure 3A j).

In addition, no significant difference in NSE was observed in testicular tissues between normal and cryptorchid testes in Bactrian camels, and both the SP and NFH average absorbance were increased in cryptorchid testicular tissues.A much higher abundance of DβH was observed in the cryptorchid group than in the normal group (Figure 3B).

Expressions of neuroendocrine markers were further examined in normal and cryptorchid testes of Bactrian camels by western blotting. As shown in Figures 3C and D, the protein levels of NFH and DβH were significantly increased in cryptorchid testicular tissues (*p*<0.01).

### Comparisons of the localization of NSE, SP, NFH, and DβH between normal and cryptorchid testes of Bactrian camels.

**Figure 4:**
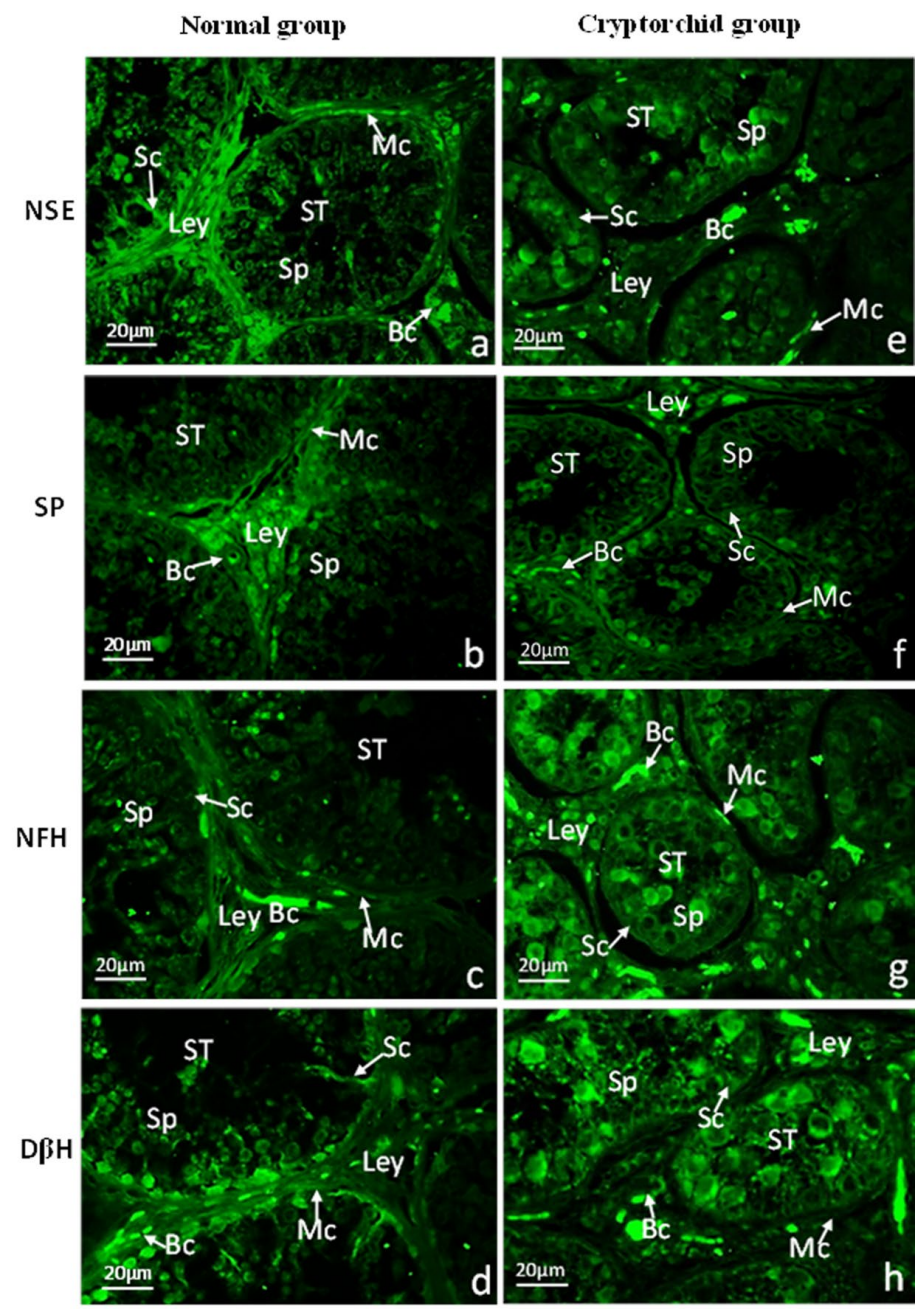
Localization of NSE,SP,NFH and DβH in the normal and cryptorchid testes of Bactrian camels. Green color: Immunofluorescence representing the reaction of antibodies with antigens; a-d: Normal Bactrian camels; e-h: Cryptorchid Bactrian camels; (a,e): stained with NSE polyclonal antibody; (b, f): stained with SP polyclonal antibody; (c, g): stained with NFH: (d, h) stained with DβH polyclonal antibody; Blood capillary (BC); Leydig cells (Ley); Myoid cells (Mc); Sertoli cells (Sc); Spermatocytes (Sp); Seminiferious tubule(ST).scale bar=20μm.

Green immunofluorescence analysis indicated that NSE was mainly expressed in Leydig cells and weakly expressed in the seminiferous epithelium (Sertoli cells, peritubular myoid cells and spermatogenic cells)of the normal testicular tissues (Figure 4a), and SP was prominently expressed in Leydig cells and nerve fibers in interstitial tissue (Figure 4b). NFH was weakly stained in Leydig cells of normal testes (Figure 4c). Similarly, DβH was weakly expressed in Leydig cells, whereas intense staining was detected in Sertoli cells, and no expression was detected in peritubular myoid cells of the normal group (Figure 4d; Table 2).

**Table 2:** Expression intensity of NFH, SP,NSE and DβH in different cells of the normal and cryptorchidism of Bactrian camel testis.

**Group**	**Sertoli cells**	**Spermatocytes**	**Leydig cells**	**Peritubular myoid cells**	**Endothelial cells**
NSE-Normal group	++	+	++++	++++	++++
NSE-Cryptorchid group	+	+	++	++	+++
SP-Normal group	+	-	+++	++	++
SP-Cryptorchid group	+	+ -	++	-	-
NFH-Normal group	-	-	-	-	++++
NFH-Cryptorchid group	+	++	+	+	+++
DβH-Normal group	++++	+	-	+++	+++
DβH-Cryptorchid group	+++	++	-	-	+++

The intensity of immunofluorescence results was scored as follow: -: no positive expression; +/-: occasional positive expression; +: positive expression; ++: medium-intensity positive expression; +++: strong positive expression; ++++: high-density strong positive expression (Amadio et al., 2007).

As shown in Figure 4e-f, , weak staining of NSE was observed in the whole testicular tissues of cryptorchid tissues (Figure 4e), and SP was weakly expressed in the interstitial tissue (Figure 4f).NFH protein was highly expressed in cytoplasm-rich primary spermatocytes and Leydig cells, with occasional expression observed in atypical nuclear-shaped Sertoli and peritubular myoid cells (Figure 4g). DβH protein was strongly stained in the Sertoli and spermatogenic cells,while no expression was observed in peritubular myoid cells or Leydig cells (Figure 4h; Table 2).

## Discussion

Cryptorchidism is often associated with testicular heat stress, leading to pathological changes, including decreased sperm motility, delayed development of adult stem cells, meiotic disorders, and reduced number of germ cells (Ali et al., 2014; Pinart et al., 2000).Cryptorchidism of six-month-old Ziwuling Black goats caused a reduction in the number of spermatogenic epithelial cells, a significant decrease in the average diameter of the seminiferous tubules, abnormal differentiation of Sertoli cells, and retarded sperm development in the testes (Yuan et al., 2021). Male Bactrian camels reach sexual maturity at approximately four years old,and breeding starts at five. The age of Bactrian camels studied here was two-year-old had not reached mating age yet. The seminiferous tubules were developed with three to five layers of seminiferous cells,but lack of obvious sperm in the seminiferous epithelia. Moreover, the cryptorchidism caused a reduction in layers of spermatogenic epithelium, which only comprised one to three layers. Therefore, the e following experiments were designed to explore the underlying mechanisms of spermatogenic defects in cryptorchidism.

The interstitial connective tissue of mammalian testes is composed of different ingredients, such as blood capillaries, lymphoid vessels and Leydig cells. Leydig cells in testicular sections play crucial roles in male development and maintaining reproductive functions (Zhuang et al., 2004). We have demonstrated that the swelling of mitochondria in Leydig cells was related to oxidative stress in cryptorchid yaks (Chen et al., 2015). Novo et al. (2021) revealed that oxidative stress mediates mitochondrial dysfunction and programmed cell death.In the present study,the villus protrusions of Leydig cells were not prominent and with swelling mitochondria and indistinct reticulum crowding in the cytoplasm of Leydig cells in cryptorchidism.A previous study focused on the similarities between aging-associated and cancer-associated oxidative stress and mitochondrial dysfunction as their typical phenotype (Kudryavtseva et al., 2016). In our study,the pathological changes of cryptorchidism in camels should be closely related to the decrease in oxidative capacity of swollen mitochondria in Leydig cells.

There are two distinct populations of Leydig cells,fetal and adult,that arise at different times during the development of the testis (Martin, 2016). The adult population, appearing at or just before puberty,is responsible for spermatogenesis and maintaining male secondary sex characteristics (Martin, 2016). Leydig cells are first recognized morphologically two days after birth with the appearance of lipid droplets in the cytoplasm of specific interstitial cells. The lipid content closely matches the steroid content of the developing testis and marks the maturation of the steroid synthesis pathway in the tammar testis (Butler et al., 2008). Research has shown that the minimal testosterone concentration in testicular tissues occurs in 2-year-old camels during the nonbreeding season,which was lower than that in 1.5-year-old and 3-year-old young camels and then increased with advancing age (El-Harairy and Attia, 2010).

Furthermore, the onset of puberty coincides with a dramatic increase in the average Leydig cell size, accompanied by a peak in the steroid-producing capacity per Leydig cell (Pasha et al., 2011).The present results show that the lipid droplets in Leydig cells were non-significant in cryptorchidism tissue compared to the normal camel, which was still at the early stage of pre-pubertal and sexual activity was not obvious and Leydig cells were in the process of development. Much evidence suggested that there is a significant correlation between the testosterone concentration and the size and number of Leydig cells (Curley et al., 2019; Mularoni et al., 2020).Our results showed that the average area of Leydig nuclei in cryptorchid tissues was significantly lower than that in normal testes. It was consistent with that of cryptorchid goats (Yuan et al., 2021). Ezeasor (1985) demonstrated that the increase of the accumulation of hormone precursors in Leydig cells in cryptorchid goats stimulates immune stress due to decreases in the synthetic activity of Leydig cells. Therefore, our findings regarding the ultrastructural alterations in the Leydig cell indicate defects in the steroid synthesis pathway in cryptorchidism, and also we observed that cryptorchidism caused obvious phagocytic characteristics of the macrophages near Leydig cells,it may be an clinical cue for degraded testes immune microenvironmental system.

Glycoproteins are critical biological macromolecules present at the cell membrane, intercellular substance, plasma, and mucus. Glycogen and glycoprotein are abundantly expressed in neurons,endocrine,and neuroendocrine cells (Buckley and Kelly, 1985; Moore et al., 2010; Terzano et al., 2012). Prior studies showed that the positive bands of PAS were primarily distributed in the vascular wall and the basement membrane of seminiferous tubules in the testes of dromedary camels. It was indicated that the camel testis contains a wide range of glycoconjugates and specific carbohydrate structures are required for spermatogenesis during periods of the rutting season and are particularly apparent in the breeding period (Osman et al., 1976; Abd-Elmaksoud et al., 2008).Methods for detecting mucin are considered with the AB staining; acidic mucus containing hydroxyl and sulfate groups forms an insoluble complex that stains blue (Culling, 1974; Walsh and Jass 2000). The levels of acidic mucus increase with the establishment of the Leydig cell groups and the maturity of spermatogenic cells (Moore et al., 2010).Our previous study observed PAS-positive bands distributed in the vascular wall and lamina propria of seminiferous tubules in the normal goat testes, while cryptorchidic goats caused a decrease in glycoproteins,which may indicate that saccharides function as essential components of primary nutrition metabolism,and play crucial roles in spermatogenesis (Yuan et al., 2021). Here, we observed the same results in the cryptorchidic Bactrian camel. The sulfated glycoproteins protect the normal activity of sperm (Liu, 2016).Our studies revealed that the cryptorchidism decreased the diameter of the Leydig cell (*p*< 0.05) and induced glucose metabolism disorders in the surrounding connective tissue, which may contribute to the abnormal distribution of local neurohormones in testis.

Soluble brain protein 14-3-2, first described by Moore and McGregor in 1965, is now known as a cell-specific isoenzyme of the glycolytic enzyme enolase, designated neuron-specific enolase (NSE) (Moore and Mcgregor, 1965). It is not only a marker for all types of neurons,but also for all neuroendocrine or paraneuronal cells (Schmechel et al., 1978). It has been proven that Leydig cells of human testis possess neuroendocrine properties and are therefore a member of the diffuse neuroendocrine (paraneuron) system, and NSE is immunopositive in the cytoplasm of human Leydig cells (Schulze et al., 1991). Comparative immunocytochemical studies have demonstrated that NSE was detected in Leydig cells of the golden hamsters, guinea pigs, and rats at all stages studied: fetal, neonatal and adult. NSE may be synthesized by Leydig cells and synthesized steroids (Angelova et al., 1991; Schulze et al., 1991).

Furthermore, the expression intensity of NSE in the testes of young rats and hamsters was higher than that of adults, and NSE is positively expressed not only in Leydig cells,but also in sperm cells and Sertoli cells in adult guinea pig testes (Angelova et al., 1991).The demonstration that the nervous system and neuroendocrine origin tumors contain NSE promoted the NSE as a possible tumor marker. Immunohistochemical staining revealed positive expression of NSE in human testicular tumors (Lu et al., 2015). NSE was predominantly detected in Sertoli cells caused by Leydig cell tumors in canine cryptorchidism (Owston and Ramos-vara, 2007). Here, the expression of NSE was only observed in Leydig cells and vascular endothelial cells of normal testes. More notably, NSE was expressed not only in Leydig cells but also in Sertoli cells in cryptorchidism. Although there was no significant difference between the two groups, this difference in NSE expression is helpful to judge the pathological changes of testicular tumors. Our results suggested that cryptorchidism of the Bactrian camel tends to malignant transformation.

SP was accidentally isolated from the intestinal plain muscle and brain of horses in the 1930s by Euler and Gaddum (1931), which lowers arterial blood pressure and stimulates the tone and rhythm of the rabbit's isolated intestine. Euler (1934) demonstrated that human semen and extracts of the prostate glands of various animals have pharmacological actions due to SP. SP immunoreactivity can be detected in Leydig cells, particularly human testes, and to a lesser degree in mouse Leydig cells, it could be potentially paracrine substance regulating intratesticular function (Chiwakata et al., 1991). SP is present in Leydig and Sertoli cells of humans, mice, hamsters, marmosets, and rats, and evidence suggests that it has potential regulatory roles regarding spermatogenesis, spermatozoa function and motility, and testicular steroidogenesis (Blasco et al., 2019).The SP immunopositive fibers in camel testes were mainly distributed in the caput and cauda of testes (Saleh et al., 2002). Expression of SP in cremaster muscle was decreased in cryptorchid tissues, resulting in a decrease in testicular autonomic nerve regulation, and testicular retention in the groin (Tanyel et al., 2001). Our study showed that immunostaining of SP in Leydig cells and peripheral nerve fibers in normal testes was strongly positive; however, its distribution in cryptorchid tissues was significantly decreased. Therefore, our results suggest the distribution differences of SP in cryptorchidism may reduce the local autonomic innervation and be related to steroidogenesis of the Bactrian camel.

Neurofilaments (NFs) are composed of light (NF-L), medium (NF-M),and heavy(NF-H)subunits of approximately 68,145, and 200 kDa, respectively. All three subunits are phosphorylated and most of the phosphorylation sites are located in the tail domain of NFH (Laser-Azogui et al., 2015) New evidence that NFH exists within axons and influences neurotransmission suggests that NFH might contribute to normal synaptic function and neuropsychiatric disease states (Yuan et al., 2017a). A correlation was found between the expression of NFH immunoreactivity in Sertoli cells and the stage of spermatogenesis; in human cryptorchidism, Sertoli cells exhibited strong NFH immunoreactivity whereas Sertoli and Leydig cells showed no or only weak reactivity in normal testes (Davidoff et al., 1999). NFH immunoreactivity in perivascular locations, intermingled with interstitial cells and close to the wall of seminiferous tubules was found only in the testes from immature rhesus monkeys.

Furthermore,it shows a marked degree of plasticity during development, especially around the time of puberty, and in cells of the tubular wall and vascular cells directly and/or indirectly via the intermediation of mast cells (Frungieri et al., 2000). Immunohistochemical analyses showed that expression of NFH has significantly increased in Sertoli cell only syndrome and germ cell arrest biopsies in males (Mayerhofer et al., 1999).The results showed that during the recovery of rat testicular Leydig cells following ablation with ethane dimethanesulfonate,expression of NFH in Leydig cells increased,suggesting that NFH contributes to the proliferation of Leydig cells (O'Shaughnessy et al., 2008). We also discovered that immature Leydig cells, the abnormal proliferation of the Sertoli cells and arrested spermatogenesis were the main characteristics in cryptorchid testes of Bactrian camels (Müller et al., 2006; Yuan et al., 2017b). The present study provides the first evidence at the protein level for the existence of NFH in Sertoli and Leydig cells of the Bactrian camel cryptorchidism, and western blot studies demonstrated that NFH was significantly increased in cryptorchid testicular tissues. Our study demonstrates that NFH correlates with developmental abnormalities of Sertoli and Leydig cells in Bactrian camel cryptorchidism.

Dopamine β-hydroxylase (DβH) is an adrenergic monoamine enzyme. DβH immuneoreactivity in the cytoplasm of interstitial Leydig cells in human testes provides strong evidence for the neuroendocrine nature of the Leydig cells (Plant, 2015). Immunohistochemical studies in pigs suggest that DβH-positive neurons should be considered elements of highly testosterone-dependent autonomic circuits involved in the regulation of urogenital function (Kaleczyc et al., 2019). A previous study identified Leydig cells as the presumed sites of catecholamine production in both fetal and mature human testes and further supported the recognized neuroendocrine characteristics of this cell type (Davidoff et al., 2005). Romeo et al. (2004) found that DβH is a neurotransmitter involved in the regulation of testicular function and activates Leydig cell receptors, especially, during psychogenic or somatic stress environments, which could contribute to the high concentration of catecholamines and suppression of testicular functions. Considering the controversial data presented in previous reports, compared to the control cases, Leydig cell-specific expression of DβH was significantly weaker in human cryptorchidism and suggested that Leydig cells are not essentially involved in the pathogenesis of the disturbances (Middendorff et al., 1993). These results are in accordance with our immunohistochemical and western blotting data for DβH, which was weakly expressed in Leydig and peritubular myoid cells in Bactrian camel cryptorchidic tissue. We suggest that DβH expression in Leydig cells has little to do with whether they are normal. However, more importantly, the results presented here provide evidence that DβH is strongly expressed in the Sertoli and spermatogenic cells, which is very different from other reports.

Sertoli cells are involved in the regulation of testis-specific differentiation processes,such as prevention of germ cell entry into meiosis, Leydig and peritubular myoid cell differentiation, regression of the Müllerian duct, and the upper part of the vagina, also hide from the host immune system and secrete trophic factors (Barrionuevo et al., 2011). Sertoli cells can be divided into three stages: embryonic Sertoli cells (eSCs), immature Sertoli cells, and mature Sertoli cells, eSCs possess multiple supporting functions and research value in gonadal development and sex determination (Yue et al., 2006; Procópio et al., 2017).In addition, the effect of GDNF secreted by Sertoli cells on the differentiation potential of dopaminergic neurons has been reported (Yue et al., 2006; Barrionuevo et al., 2011; Procópio et al., 2017). Negative expression of DβH by immunostaining and RT-PCR in human embryonic stem cells supported that the differentiated neurons induced by Sertoli cells are dopaminergic rather than noradrenergic (Procópio et al., 2017); Our study suggests that expression of DβH from Sertoli cells of Bactrian camels is very important for normal development of both testicular somatic cells and germ cells, and it may provide a promising method for therapeutic application and basic neuroscience research in the Sertoli-derived DβH neuron differentiation. The production of sperm and androgens depends on the normal development of both testicular somatic cells and germ cells, and spermatogenic cell development depends entirely on somatic cells, especially Sertoli cells (Mäkelä et al., 2019). Cryptorchidism can occur due to malfunctions in somatic and germ cells (Mäkelä et al., 2019). In cryptorchid patients, the diminished number and functional defects of Sertoli cells prove its pivotal role in germ cell development (Hamdi et al., 2017; Zivkovic and Hadziselimovic, 2009). In the current study, DβH expression in the cryptorchidism group was significantly higher than that in the normal group, DβH was strongly stained in the Sertoli cells,indicating a close relationship between DβH and Sertoli cell. Dysplasia of Sertoli cells and abnormal proliferation of spermatogenic epithelial cells may be one of the reasons for the increased expression of DβH. Furthermore, additional neuroendocrine characteristic studies specifically in Sertoli cells are needed to amplify our findings.

## Conclusions

The current study demonstrated that the pathological changes in cryptorchid tissue of Bactrian camels are potentially related to disorders in glycoprotein metabolism. we provides the first evidence at the protein level for the existence of NFH and DβH in Sertoli and Leydig cells in Bactrian camel cryptorchidism and indicates a close relationship between DβH and Sertoli cells. NSE and SP were correlated with developmental abnormalities of Leydig cells in Bactrian camel cryptorchidism. Therefore, our results suggest that distribution differences of NSE, NFH, SP and DβH in normal and cryptorchid testes of Bactrian camels provide a more in-depth understanding of neuroendocrine regulation that is crucial in animal cryptorchidism.
